# ZnO nanosheet arrays constructed on weaved titanium wire for CdS-sensitized solar cells

**DOI:** 10.1186/1556-276X-9-112

**Published:** 2014-03-11

**Authors:** Cuncun Wu, Lin Wei, Yitan Li, Chang Liu, Jun Jiao, Yanxue Chen, Liangmo Mei

**Affiliations:** 1 School of Physics and State Key Laboratory of Crystal Materials, Shandong University, Jinan 250100, People's Republic of China; 2 School of Information Science and Engineering, Shandong University, Jinan 250100, People's Republic of China; 3 Department of Mechanical and Materials Engineering, Portland State University, P.O. Box 751, Portland, OR 97207-0751, USA

**Keywords:** ZnO nanosheets, CdS, Nanoparticles, Solar cells

## Abstract

Ordered ZnO nanosheet arrays were grown on weaved titanium wires by a low-temperature hydrothermal method. CdS nanoparticles were deposited onto the ZnO nanosheet arrays using the successive ionic layer adsorption and reaction method to make a photoanode. Nanoparticle-sensitized solar cells were assembled using these CdS/ZnO nanostructured photoanodes, and their photovoltaic performance was studied systematically. The best light-to-electricity conversion efficiency was obtained to be 2.17% under 100 mW/cm^2^ illumination, and a remarkable short-circuit photocurrent density of approximately 20.1 mA/cm^2^ was recorded, which could attribute to the relatively direct pathways for transportation of electrons provided by ZnO nanosheet arrays as well as the direct contact between ZnO and weaved titanium wires. These results indicate that CdS/ZnO nanostructures on weaved titanium wires would open a novel possibility for applications of low-cost solar cells.

## Background

Since the first work pioneered by O'Regan and Grätzel in 1991, dye-sensitized solar cells have been investigated extensively during the past two decades as promising alternatives to conventional silicon solar cells [1-5]. Although the light-to-electric conversion efficiency of 12% [6] reported recently was very impressive, the use of expensive and instability dyes to sensitize the solar cell is still not feasible for practical applications. Therefore, it is critical to tailor the materials to be not only cost-effective but also long lasting.

Narrow bandgap semiconductor nanoparticles, with unique bandgap characters, have been put forward as an efficient and promising alternative to ruthenium complexes or organic dyes in solar cell applications. Compared with conventional dyes, semiconductor nanoparticles have several advantages: (i) The large intrinsic dipole moments of the nanoparticles can lead to a large extinction coefficient and rapid charge separation, which is known to reduce the dark current and increase the overall efficiency. (ii) Because of their strong quantum confinement effect, the bandgap of semiconductor nanoparticles can be tuned by their sizes to match the solar spectrum. (iii) Furthermore, multiple exciton generation, where an electron with sufficiently high kinetic energy can generate one or more additional electron–hole pairs, has been predicted in semiconductor nanoparticles, providing new chances to utilize hot electrons or generate multiple charge carriers with a single photon. Hence, nanosized narrow bandgap semiconductor nanoparticles are promising light absorbers for solar cells to achieve improved performance.

A range of nanosized semiconductors, including CdSe [7-9], CdS [10-12], PbS [13,14], and Cu_2_O [15], have been studied as sensitizers in place of conventional dye molecules for solar cell applications. For most of the reported nanostructured solar cells, transparent conductive oxide (TCO) glass is used as the substrate material. It is fragile, heavyweight, and a little high resistive, hampering its application in large-area solar cell modules. Recently, flexible solar cells, which are lightweight, portable, and economically cheap, have attracted significant academic interest and industrial attention. Indium tin oxide (ITO)- or fluorine-doped tin oxide (FTO)-coated polymer substrates are widely used as the substrate for flexible solar cells. However, the low temperature tolerance of those flexible plastic substrates limits the solar cell preparation process only below 200°C, resulting in a poor crystallization and photovoltaic performance. Metals with good flexibility, low resistance, high-temperature sinterability, and low cost are promising candidates as substrates in lightweight solar cells. Among the metals, Ti metal substrate, which has superior corrosion resistance to electrolytes in sensitized solar cells, has been studied by many groups [16-20]. It is expected that the application of weaved titanium wires as support of TiO_2_ or ZnO can not only reduce the weight of solar cell but also contribute to improve the performance of the solar cells by reducing internal resistance. However, most of the published works were based on conventional organic dyes; little work has been carried out on inorganic nanoparticles.

In this paper, ordered ZnO nanosheet arrays were grown on weaved titanium wires using a low-temperature hydrothermal method. By a successive ionic layer adsorption and reaction (SILAR) method, CdS nanoparticles were deposited onto the ZnO nanosheet arrays to fabricate CdS/ZnO nanostructures as a photoanode for a practical nanostructured solar cell. The effect of CdS SILAR cycles on the photovoltaic performance was studied systematically, and the optimized solar cells show a best light-to-electricity conversion efficiency of 2.17% with a short-circuit current density of 20.1 mA/cm^2^.

## Methods

### Preparation of ZnO nanosheets on titanium substrate

ZnO nanosheets were prepared by a two-step simple chemical solution method. In the first step, a layer of ZnO seeds was deposited onto weaved titanium wires by dipping the mesh in an alcohol solution containing 0.02 M zinc acetate dihydrate and 0.02 M lithium hydroxide, followed by annealing in a furnace at 400°C for 1 h. Then, the seeded substrate was placed into a glass bottle which contains an aqueous solution with 0.2 M of zinc nitrate and 1 M of urea. In the second step, the hydrothermal growth was conducted by heating the solution to 90°C for 12 h. After the hydrothermal treatment, the resultant nanostructure was rinsed with deionized water thoroughly and then annealed at 450°C for 1 h to remove any residual organics and convert into ZnO nanosheets.

### Deposition of CdS nanoparticles with successive ionic layer adsorption and reaction method

CdS nanoparticles were deposited onto the ZnO nanosheet surface by SILAR method. Solutions of 0.05 M cadmium nitrate (Cd(NO_3_)_2_) and 0.05 M sodium sulfide (Na_2_S) were prepared by dissolving Cd(NO_3_)_2_ in deionized water and Na_2_S in methanol/water with volume ratios of 1:1. In a typical SILAR cycle, weaved titanium wire substrate, pre-grown with ZnO nanosheet arrays, was dipped into the Cd(NO_3_)_2_ aqueous solution for 30 s, rinsed in water, then dipped into the Na_2_S solution for another 30 s, and rinsed again in ethanol. This entire SILAR process was repeated to achieve the desired thickness of CdS nanoparticles. After the synthesis, the CdS/ZnO/Ti substrate was carefully washed in deionized water and dried at 100°C.

### Characterization

The morphologies of the ZnO/Ti and CdS/ZnO/Ti nanostructures were examined using a field-emission scanning electron microscope (FESEM; FEI Sirion, FEI Company, Hillsboro, OR, USA). The crystal structures of ZnO/Ti and CdS/ZnO/Ti were examined by X-ray diffraction (XRD; XD-3, PG Instruments Ltd., Beijing, China) with Cu Kα radiation (*λ* = 0.154 nm) at a scan rate of 2°/min. X-ray tube voltage and current were set at 40 kV and 30 mA, respectively. The optical transmission spectra were obtained using a dual-beam UV-visible spectrometer (TU-1900, PG Instruments Ltd., Beijing, China).

### Solar cell assembly and performance measurement

**Figure 1:**
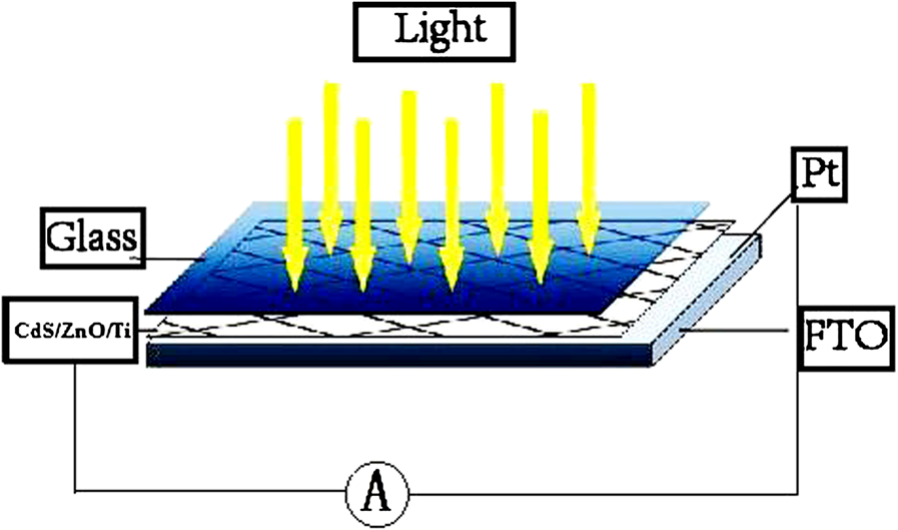
Schematic diagram of the CdS/ZnO/Ti nanostructured solar cell.

The schematic structure of the nanostructured solar cell is shown in Figure 1. The solar cell was assembled using the CdS/ZnO/Ti nanostructure as the photoanode and a platinum-coated FTO glass as the counter electrode. The counter electrode was prepared by spin coating a solution of H_2_PtCl_6_ (0.01 M) in isopropyl alcohol on FTO glass and subsequently annealed it at 500°C for 30 min. A 60-μm-thick sealing material (SX-1170-60, Solaronix SA, Aubonne, Switzerland) with a 4 × 4 mm^2^ aperture was sandwiched between the titanium mesh substrate and the counter electrode to prevent electrical shorts. A polysulfide electrolyte was injected into the space between the two electrodes. The polysulfide electrolyte was composed of 1 M sulfur, 1 M Na_2_S, and 0.1 M NaOH, all of which were dissolved in deionized water and stirred at 80°C for 2 h.

The photovoltaic performance was characterized under an AM 1.5 G filter at 100 mW/cm^2^ using a Newport Oriel 94022A Solar Simulator (Model 94022A, Newport, OH, USA), as calibrated using a certified OSI standard silicon photodiode. A sourcemeter (2400, Keithley Instruments Inc., Cleveland, OH, USA) was used for electrical characterization during the measurements.

## Results and discussion

### Morphology and crystal structure of the nanostructured photoanodes

**Figure 2:**
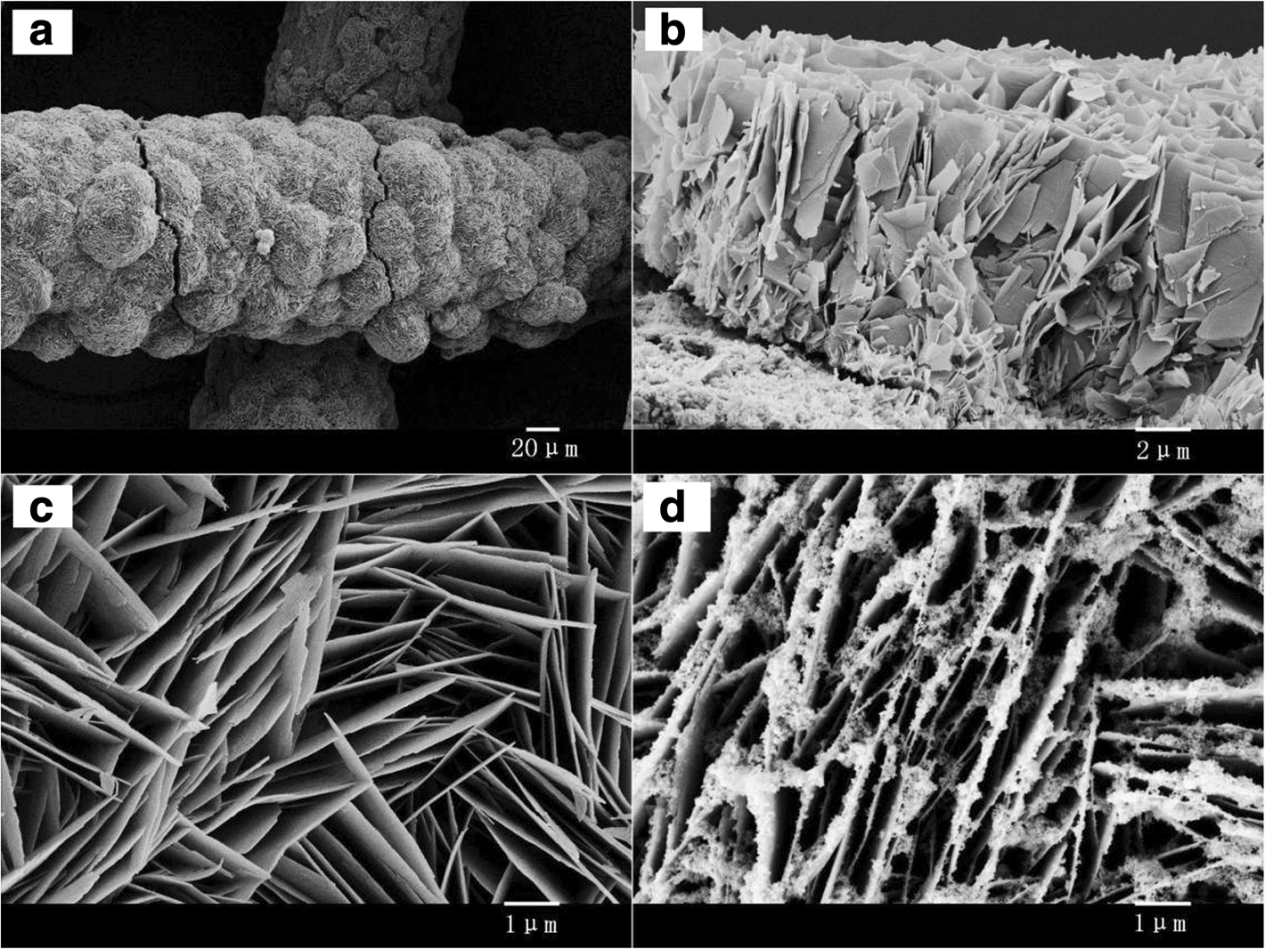
**Typical FESEM images of ZnO nanosheets on weaved titanium wire substrate. (a)** The low-magnification and **(c)** high-magnification FESEM images of ZnO nanosheets. **(b)** The cross-sectional view of ZnO nanosheets. **(d)** ZnO nanosheets deposited with CdS nanoparticles for 20 cycles.

The employed weaved titanium wire is flexible and of a diameter of about 85 μm with quite smooth surface. The color of the weaved titanium wire changed from gray to white after the deposition of ZnO nanosheets. Figure 2a shows the typical FESEM images of ZnO nanosheet arrays grown on weaved titanium wires. The surface of the titanium cylinder wires is covered totally and uniformly with ZnO nanosheet arrays, which would provide a large area for the deposition of CdS nanoparticles. Figure 2b shows the cross-sectional SEM image of ZnO nanosheets. It is apparent that all products consist of a large number of well-aligned sheet-like nanostructures. The SEM image clearly indicates that the film is constructed by assembling nanosheets in a compact way and the nanosheets are vertically oriented to the surface of titanium wires with different angles to each other. The average film thickness is about 8 to 10 μm. Figure 2c,d shows the top view of the ZnO nanosheets and CdS/ZnO nanostructures at a high magnification, respectively. The space between nanosheets presents an easily accessed open structure for the deposition of CdS nanoparticles, which is very important for the performance of solar cells. Furthermore, this open structure could provide an easy filling of electrolyte into the space between the nanosheets and is beneficial to hole diffusion from CdS nanoparticles to counter electrode. By comparing Figure 2c,d, it can be clearly seen that the CdS nanoparticles were uniformly deposited onto ZnO nanosheets. The CdS nanoparticles make direct contact with the ZnO nanosheet surface, forming a firm connection on the ZnO nanosheets with a type II heterojunction, which may greatly enhance charge transport, charge separation, and overall photocurrent efficiency of the solar device.

**Figure 3:**
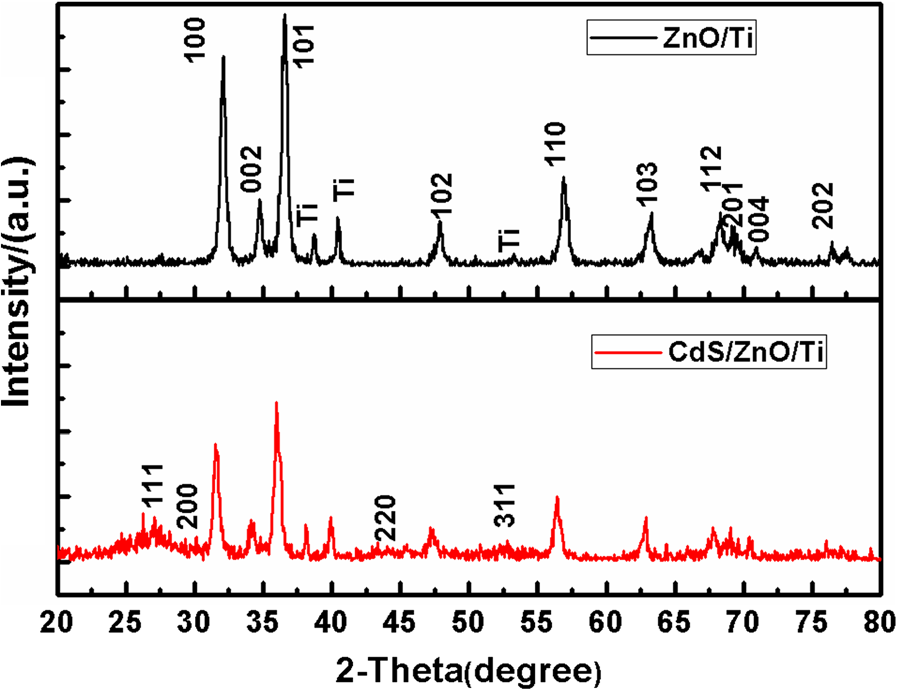
XRD patterns of ZnO nanosheets (black line) and ZnO/CdS nanosheets on weaved titanium wires (red line).

XRD patterns of ZnO/Ti and CdS/ZnO/Ti nanostructures are shown in Figure 3. Note in Figure 3 (black line) that, with the exception of the diffraction peaks from the weaved titanium wire, all the other diffraction peaks could be indexed as the (100), (002), (101), (102), (110), (103), (112), (201), and (004) planes of hexagonal wurtzite crystal structure of ZnO (JCPDS 80–0074). The strong and narrow diffraction peaks demonstrate good crystallinity. No appearance of other diffraction peaks indicates the high phase purity. The XRD pattern of CdS-sensitized ZnO nanosheets after 20 cycles is also shown in Figure 3 (red line). It is observed that the CdS/ZnO nanostructure exhibits weak diffraction peaks at 2*θ* = 26.56°, 30.74°, 44.05°, and 52.11°, corresponding to the (111), (200), (220), and (311) planes, respectively, of CdS cubic phase crystal structure (JCPDS 80–0019). This result confirms the successful deposition of CdS nanoparticles on ZnO nanosheet arrays.

### Optical property of the CdS nanoparticles

**Figure 4:**
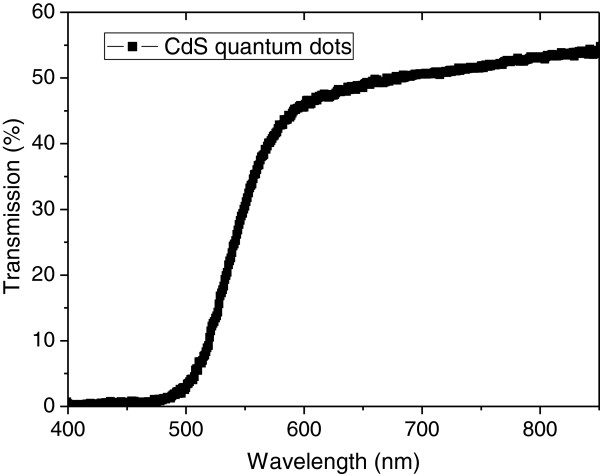
Typical optical transmission spectrum of CdS/ZnO nanostructures.

The UV-visible transmission spectrum of CdS/ZnO nanostructure sample was recorded using a ZnO nanosheet array without CdS nanoparticles as the reference. As shown in Figure 4, an optical bandgap of 2.4 eV is estimated for the as-synthesized CdS nanoparticles from the transmission spectrum, which is close to the bandgap of bulk CdS. No obvious blueshift caused by quantum confinement is observed, indicating that the size of the CdS grains is well above the CdS Bohr exciton diameter (approximately 2.9 nm). A strong absorption was observed for light with a wavelength shorter than 540 nm, corresponding to the most intensive part of the solar spectrum.

### Photovoltaic performance of the solar cell based on CdS/ZnO/Ti nanostructures

**Figure 5:**
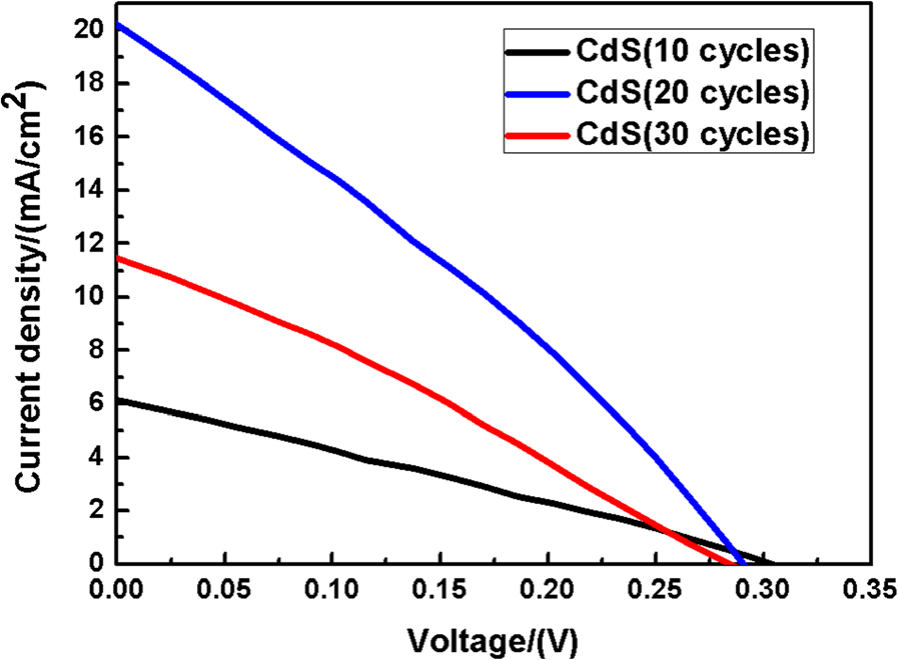
***I*-*V *** **curves for CdS/ZnO/Ti nanoparticle-sensitized solar cell with different CdS SILAR cycles.**

Figure 5 shows the photocurrent-voltage (*I*-*V*) performance of the sensitized solar cells assembled using CdS/ZnO/Ti nanostructured photoanodes. The *I*-*V* curves of the samples were measured under 1 sun illumination (AM1.5, 100 mW/cm^2^). All the photocurrent-voltage performance parameters are summarized in Table 1. Figure 5 depicts the correlation between SILAR cycles and performance parameters obtained from CdS/ZnO/Ti nanostructured solar cells. As the SILAR cycles increase from 10 to 20, more CdS nanoparticles are deposited onto the ZnO nanosheets, the *J*_sc_ and the *V*_oc_ of the solar device increase correspondingly. The best *J*_sc_ of 20.1 mA/cm^2^ is obtained for the sample with 20 SILAR cycles, indicating a light-to-electricity conversion efficiency of 2.17%. This remarkable short current density could be ascribed to the direct contact between ZnO and weaved titanium wires with low internal resistance, which provided a more desirable pathway for electron transport. When the SILAR cycles further increased, the conversion efficiency of the solar cell decreased. This decrease could be attributed to the increasing thickness of the CdS layer, which largely increases the resistance in solar cells and blocks the pathway for electrons from the photoanode to the weaved titanium wire.

**Table 1:** ***V***_**oc**_**, *J***_**sc**_**, FF, and efficiency**

	***V***_**oc **_**(V)**	***J***_**sc **_**(mA/cm**^**2**^**)**	**FF (%)**	***η *** **(%)**
CdS-10 cycles	0.31	6.1	0.32	0.61
CdS-20 cycles	0.29	20.1	0.37	2.17
CdS-30 cycles	0.28	11.4	0.34	1.10

*V*_oc_, open-circuit voltage; *J*_sc_, short-circuit photocurrent density; FF, fill factor; *η*, energy conversion efficiency.

Our findings suggest the possible use of narrow bandgap semiconductor nanoparticles grown by simple SILAR method and inorganic semiconductor nanostructure material grown by a facile hydrothermal method for sensitized solar cell application. The CdS/ZnO nanostructures on weaved titanium wires can also be used as the photoanode in low-cost, flexible sensitized solar cells. In the present work, the power conversion efficiency of our solar cells was still not high enough for the practical applications. The rather poor fill factor is considered to be the main factor limiting the energy conversion efficiency. This low fill factor may be caused by the lower hole recovery rate of the polysulfide electrolyte, which leads to a higher probability for charge recombination [21]. To further improve the efficiency of these nanosheet array solar cells, some new hole transport medium must be developed, one with suitable redox potential and low electron recombination at the semiconductor and electrolyte interface. Counter electrodes have also been reported to be another important factor influencing the energy conversion efficiency. Recently, a number of novel materials have been examined and tested as counter electrode materials; these studies prove the influence of various counter electrode materials on the fill factors of solar devices [22,23]. Also, the open-circuit voltage can be further improved by using more efficient combination of semiconductor nanoparticles.

## Conclusion

In summary, we have prepared CdS/ZnO nanostructures on weaved titanium wires by a hydrothermal treatment and a SILAR method. The resultant ZnO nanostructures consisted of a large number of well-aligned nanosheets, which are oriented vertically to the surface of titanium wires. This open-structured nanosheet array is beneficial to the deposition of CdS nanoparticles. An overall light-to-electricity conversion efficiency of 2.17% was achieved under 100 mW cm^-2^ illumination for the solar cells based on CdS/ZnO nanostructures with 20 CdS SILAR cycles. This results demonstrated that weaved titanium wires could be a valid alternative to classical FTO or ITO substrate with relatively low cost and satisfied internal resistance. In addition, the application of all inorganic semiconductors on weaved titanium wires may act as a novel architecture with lower cost and effective performance for further development of nanoparticle-sensitized solar cells.

## Competing interests

The authors declare that they have no competing interests.

## Authors’ contributions

CW carried out the preparation of ZnO/CdS nanostructure samples, assembled the solar cell devices, and drafted the manuscript. YL conducted the optical absorption spectra. LW carried out the photovoltaic performance measurements. CL carried out the XRD measurements and the SEM characterization. YC supervised this work. LM and JJ analyzed the results and finalized the manuscript. All authors read and approved the final manuscript.
